# Age-dependent variation in cytokines, chemokines, and biologic analytes rinsed from the surface of healthy human skin

**DOI:** 10.1038/srep10472

**Published:** 2015-06-02

**Authors:** Patrick M. Kinn, Grant O. Holdren, Brittney A. Westermeyer, Mousa Abuissa, Carol L. Fischer, Janet A. Fairley, Kim A. Brogden, Nicole K. Brogden

**Affiliations:** 1 Division of Pharmaceutics and Translational Therapeutics, Department of Pharmaceutical Sciences and Experimental Therapeutics, College of Pharmacy, The University of Iowa, Iowa City, IA 52242, USA; 2 Dows Institute for Dental Research, College of Dentistry, The University of Iowa, Iowa City, IA 52242, USA; 3 Department of Dermatology, College of Medicine, The University of Iowa, Iowa City, IA 52242, USA; 4 Department of Periodontics, College of Dentistry, The University of Iowa, Iowa City, IA 52242, USA

## Abstract

In the skin, aging is associated with overall epidermal thinning, decreased barrier function, and gradual deterioration of the epidermal immune response. However, the presence and role of cytokines, chemokines, and biologic analytes (CCBAs) in immunosenescence are not known. Here we identified age-related changes in skin properties and CCBAs from stratum corneum of healthy human subjects, providing a means to utilize CCBAs as benchmarks for aging skin health. Transepidermal water loss and a(*) (skin redness) decreased in an age-dependent manner, and were significantly lower (p < 0.05) in Groups 2 (56.6 ± 4.6 years) and 3 (72.9 ± 3.0 years) vs. Group 1 (24.3 ± 2.8 years). In skin wash fluid, 48 CCBAs were detected; seven were significantly lower (p < 0.05) in Groups 2 and 3: EGF, FGF-2, IFNα2, IL-1RA, HSA, keratin-6, and involucrin; cortisol was significantly higher (p < 0.05) in Groups 2 and 3. Our results correspond with the pro-inflammatory shift that occurs with immunosenescence and also provides basis for understanding the inflammatory changes in normal aging skin.

The skin undergoes a number of well-known changes in structure and morphology with age, resulting in a thinning epidermis, decreased dermal vascularity, and marked cellular heterogeneity^1^. The barrier function of the skin begins to fail, and a reduced rate of epidermal barrier repair is observed after approximately age 55 when the skin surface pH starts to become less acidic^2^. Notable changes in immune function also occur with advancing age, causing a pro-inflammatory state and a shift towards dominance of Th2–mediated processes in the skin in a process known as immunosenescence^2^. Elderly patients have a diminished ability to mount a protective response against newly encountered antigens, and often do not produce sufficient antibody titers after vaccination^3^. A number of age-related changes are also observed in the type and prevalence of dermatologic pathologies. Elderly populations are affected by different dermatologic conditions than younger and middle aged populations, including an increase in contact dermatitis, eczema, pruritic dermatoses, and autoimmune disorders^4^. While the increased prevalence of these conditions is likely related to immunosenescence, the exact mechanisms and factors involved remain elusive.

Cytokines, chemokines, and biologic analytes (CCBAs) can be readily detected in plasma, urine, and saliva^5^, and expression of these CCBAs is either increased or decreased in the context of various diseases. In both normal and diseased skin, secretion of these factors can be detected on the skin surface, in sebaceous secretions, in sweat gland secretions, or in the epidermis. However, the methods used to obtain these analytes from the skin are invasive and painful (i.e., punch biopsies, suction blisters, and microdialysis). In particular, these methods are far too invasive for evaluating dermatologic conditions in elderly patients, given that aging skin displays slower rates of healing and barrier restoration after insult^1^. The lack of noninvasive methods to evaluate CCBAs significantly limits the use of this information for diagnosing skin disorders or evaluating treatment of cutaneous disease. Recent work demonstrates that CCBAs can be noninvasively rinsed from the skin surface using a simple wash technique^6^. These methods allowed detection of differences in TNFα, IL-1α, and IL-6 among human subjects with psoriasis, atopic dermatitis, and healthy skin. This suggests that pro-inflammatory CCBAs can be painlessly collected from the surface of the skin, a technique that would be particularly appropriate for evaluating skin changes and disorders in elderly patients. It also suggests that there might be differences in CCBA concentrations that correlate with age, a concept that may be especially suitable for evaluating skin immunosenescence.

While it is known that aging skin demonstrates a shift towards a pro-inflammatory state (which may also contribute to specific dermatologic disorders in the elderly), it is unknown what skin analytes are involved in the immunosenescence process. In this pilot study we hypothesized that there are age-related changes in the presence and relative concentrations of CCBAs in wash fluid of healthy aging skin. We propose that defining how the skin surface analytes change with “normal” aging will allow a point of comparison to describe how diseased elderly skin is altered in CCBA expression. This would provide a noninvasive means of identifying disease mechanisms and novel targets for treatment. However, until the changes in skin analytes as a function of normal aging are well understood, the field cannot progress forward to address the relationship of skin chemistry to skin disorders in aging populations. This is the first study to utilize a noninvasive technique to collect skin analytes from aging skin and determine quantitative differences from younger skin. Our results of this pilot study demonstrate significant age-related differences in barrier function, redness, and CCBA expression in healthy subjects.

## Results

Twenty-three subjects completed the study, and were divided into three age groups: 18–44 years (Group 1, n = 6), 45–64 years (Group 2, n = 8), and 65–95 years (Group 3, n = 9). General demographics can be seen in Table S1 in the Supplementary Material. None of the subjects had skin diseases, medical conditions, or medication use that would affect the integrity of the skin (all exclusion criteria described in the Materials and Methods section).

### Aging skin demonstrates significant differences among CCBAs

**Figure 1:**
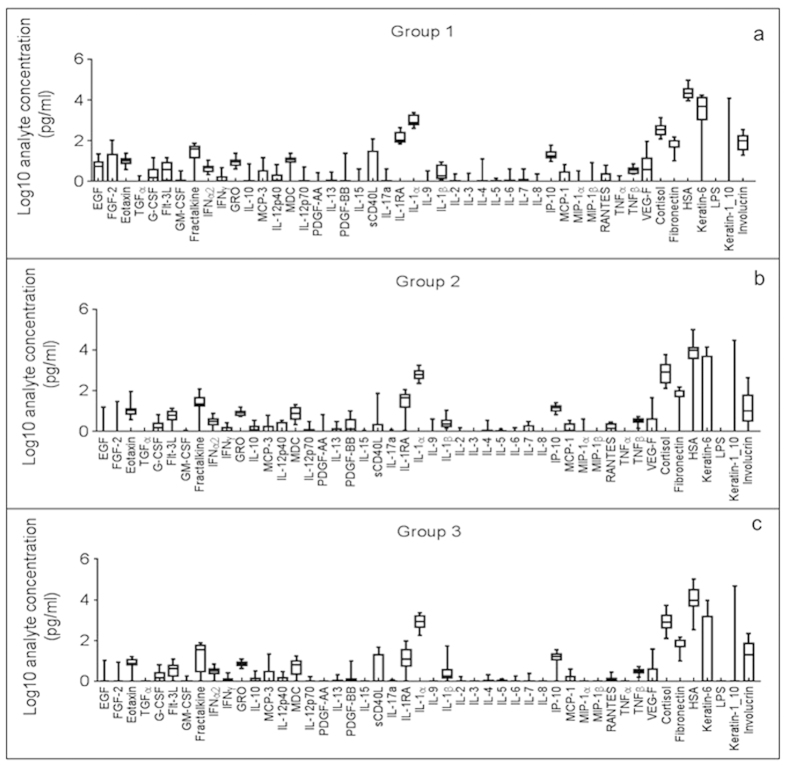
Forty-eight CCBAs were detected in skin wash fluid of healthy human subjects ranging from 21–77 years of age. Group 1 (Panel 1a): n = 6 subjects, mean ± SD age 24.3 ± 2.8 years. Group 2 (Panel 1b): n = 8 subjects, mean ± SD age 56.6 ± 4.6 years. Group 3 (Panel 1c): n = 9 subjects, mean ± SD age 72.9 ± 3.0 years. Eight CCBAs were significantly different between groups. EGF, FGF-2, IL-1RA, INFα2, HSA, keratin-6, and involucrin decreased significantly with increasing age. Cortisol increased significantly with age. The boxes represent the 25^th^ and 75^th^ percentiles, and the black line within each box represents the mean value. The upper and lower whiskers represent the maximum and minimum values, respectively.

Using a fluorescent bead-based assay, 48 CCBAs were detected in the skin wash fluid collected from three sites on the upper arm of each subject, ranging in concentration from 0–105,400 pg/ml (Table S2, Supplementary Material). Using raw values (non log-transformed data), the CCBAs fell into distinct concentration ranges: negligible (<1.0 pg/ml), low (1.0–10.0 pg/ml), moderate (10.0–100.0 pg/ml), and robust (>100.0 pg/ml). The distribution of the 48 CCBAs, based on concentration and function, can be seen in Fig. 1, Table 1, and supplementary Table S2.

**Figure 2:**
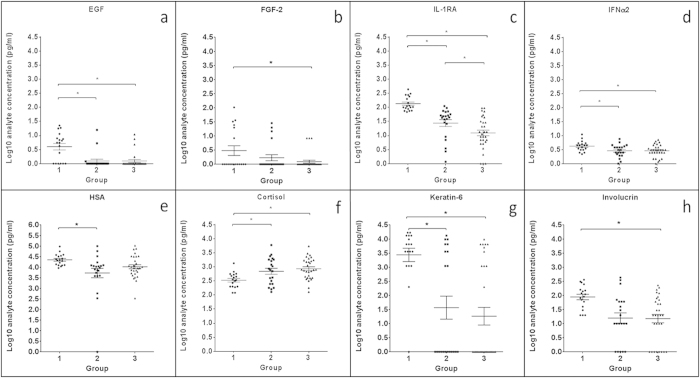
Eight CCBAs were significantly different between age groups (p < 0.05): EGF, FGF-2, IL-1RA, IFNα2, HSA, cortisol, keratin-6, and involucrin. The line represents the mean of each group, with the scatters displaying the individual data points for each group (n = 18 data points for the 6 subjects in Group 1; n = 24 data points for the 8 subjects in Group 2; n = 27 data points for the 9 subjects in Group 3). Asterisks denote significant differences between groups (p < 0.05).

Following log transformation, eight mediators were significantly different between groups (p < 0.05): EGF, FGF-2, INFα2, IL-1RA, involucrin, keratin-6, cortisol, and HSA. Of these, cortisol was the only CCBA that increased with age, with significantly higher concentrations (p < 0.05) in Group 3 vs. Group 1. The other seven CCBAs decreased overall with age. IL-1RA was significantly different between all three groups; involucrin and FGF-2 displayed significantly lower concentrations in Group 3 vs. Group 1; EGF, INFα2, and keratin-6 were significantly lower in Groups 2 and 3 vs. Group 1; HSA was significantly lower in Group 2 vs. 1. An additional two mediators trended towards significance: MDC(CCL22) and VEGF. Figure 2 displays scatter plots of the eight CCBAs that had significant differences between groups; Table 2 displays the differences between groups.

### Aging skin demonstrates altered barrier function and redness

**Figure 3:**
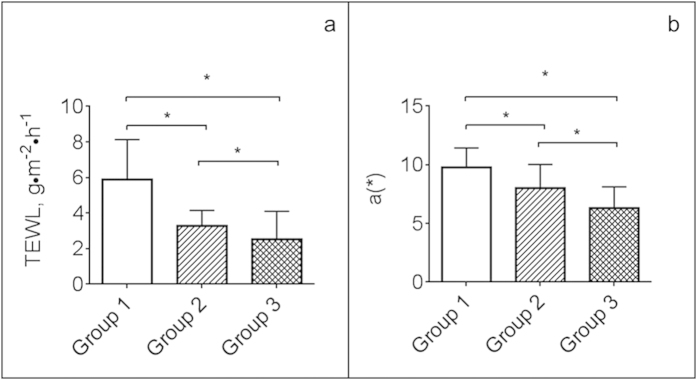
Barrier function and skin redness were significantly different between age groups. **1a**: Transepidermal water loss (TEWL) measurements, an indication of stratum corneum barrier function, decreased significantly with age. **1b**: a(*), a unitless measure of skin redness, was significantly lower with increasing age. Asterisks denote significant differences (p < 0.05).

Transepidermal water loss (TEWL) was used to quantify baseline barrier function and establish general properties of the skin at the sites where CCBAs were to be collected. TEWL measurements were made prior to collection of wash fluid in order to prevent accumulation of hydration in the skin, which would confound the data. Measurements were made in triplicate at each of three sites on the upper arm. Mean TEWL values decreased in an age-dependent manner, and were significantly lower (p < 0.05) in Groups 2 and 3 compared to Group 1 (Fig. 3). Mean ± SD TEWL measurements in Group 1 were 5.9 ± 2.3 g∙m^-2^∙h^-1^, compared to 3.3 ± 0.9 g∙m^-2^∙h^-1^ and 2.5 ± 1.6 g∙m^-2^∙h^-1^ in Groups 2 and 3, respectively.

Tristimulus colorimetry was used to quantify the redness of the skin at the sites where CCBAs were to be collected. Measurements were made in triplicate at each of three sites on the upper arm, according to previously published guidelines^7^. The values of a(*) were recorded, representing the red-green axis. Mean a(*) measurements (arbitrary units) decreased in an age-dependent manner in a trend similar to the TEWL measurements (Fig. 3). A(*) values were significantly lower in Groups 2 and 3 vs. Group 1 (p < 0.05): 9.8 ± 1.6 (Group 1) vs. 8.0 ± 2.0 and 6.3 ± 1.8 in Groups 2 and 3, respectively.

## Discussion

The stratum corneum has always been thought to be a mechanical barrier with no enzymatic or metabolic activity. More recently this concept has changed and it is now thought to be very metabolically active, producing a variety of cytokines^8^. Under both pathologic and homeostatic conditions, a number of cytokines can be identified in the skin^9^. Here, we demonstrate for the first time that many chemokines, cytokines, and other mediators can be detected on the skin surface in skin wash fluid under “normal” aging conditions using a noninvasive collection technique. Interestingly, the concentrations and profiles of CCBAs are altered in an age-dependent manner in subjects without any dermatologic disease.

TEWL measurements (used to evaluate barrier function of the skin) decreased with age, which was a somewhat unexpected finding. Skin barrier function is known to generally decline with age^10^, yet the decreasing TEWL measurements in Groups 2 and 3 suggest increasing barrier function. Still, the inverse relationship of decreasing TEWL with increasing age is consistent with what has been previously described^10^,^11^. This suggests that TEWL alone may not be sufficient for accurately describing barrier function in older populations. While gender differences in epidermal barrier function have been reported, most studies are not able to establish a difference in TEWL under basal conditions between men and women, and gender differences in TEWL resolve with advancing age^21^,^22^.

As an attempt to relate the changes in TEWL across age groups with the observed CCBA concentrations, we examined potential correlations between the TEWL values and CCBA levels. In Group 1, the correlation of TEWL with FGF-2 and involucrin was significant (p < 0.05). EGF concentrations had significant correlations with TEWL in Group 2; in Group 3 the correlation was significant for IL-1RA and HSA. This level of analysis was beyond the scope of this study (and hence should be interpreted cautiously), but these preliminary correlations point to interesting trends that could provide more insight into the interaction of specific mediators with age-related changes in epidermal barrier function.

Significantly lower a(*) values in Groups 2 and 3 vs. Group 1 (p < 0.05) were also seen, indicating decreasing skin redness. The color of the skin, especially the redness, may be in part dependent on the blood flow to the skin^12^. Blood flow to the skin decreases in an age-dependent manner^13^, which likely contributes to the age-related decreases in a(*) that we measured in our subject population.

In addition to the measurements of epidermal barrier function and redness, there were eight CCBAs that were significantly different between groups and several of these may provide very relevant information about the changing structure and function of aging skin. EGF and FGF-2 are strong inducers for the upregulation of type I procollagen production^14^, and these factors both decreased in an age-related manner in our study. While these are not the only two factors involved in type I procollagen production, decreased concentrations of these types of mediators might be somewhat expected, based on the well-known feature of decreased collagen production in aging skin. Keratin-6 is constitutively expressed at low levels in the skin and is generally known to be involved in pathways of cell proliferation, is expressed in hyperproliferative skin disorders such as psoriasis, and is induced in suprabasal cells at wound edges^15^. Our results demonstrated an age-related decrease in keratin-6, which might be in line with the impaired wound healing typically observed in aged skin. Cortisol, a glucocorticoid, was increased in the skin of our aging subjects. This could be related in a variety of ways to the adverse changes in skin morphology that are observed with aging. Glucocorticoids (endogenous or exogenous) cause undesirable effects in the skin such as dermal and epidermal thinning, reduced proliferative capacity of keratinocytes, and decreases in extracellular matrix components (including collagen)^16^; all of these are features of aging skin.

IL-1RA is an antagonist at the receptor for IL-1α, which is a pro-inflammatory cytokine. We did not observe significant age-related differences in IL-1α concentrations in our study, but IL-1RA decreased significantly with age. Hence, this finding would be supportive of the pro-inflammatory state of aging skin due to the loss of antagonism at the receptor for a pro-inflammatory cytokine. This represents an interesting concept related to the pro-inflammatory shift in aging skin: the loss of balance in the inflammatory pathways may be, at least in part, due to the loss of receptor antagonism rather than increased expression of inflammatory meditators.

IFNα2 belongs to a family of proteins produced by leukocytes that are primarily involved in innate immunity, particularly in response against viral infection. Interestingly, this analyte has also been shown to have a role in ameliorating chronic dermatitis^17^. Concentrations of IFNα2 decreased in our aging subjects, an observation that might be in good agreement with the increase in dermatitis observed in aging skin, and could possibly represent a slowly declining innate immunity that does not yet have pathologic significance.

It is only recently being described how the individuality of human subjects may alter the characteristics of the skin^18^, and our CCBA profiles suggested that this could be the case with secreted hydrophilic analytes as well. Recent work suggests that various inflammatory disease states display unique cytokine profiles, including cutaneous disorders such as psoriasis^6^,^19^,^20^. Based on a combined analysis of unique disease cytokine profiles with an individual’s CCBA signature, our methods would allow subjects to be noninvasively phenotyped at baseline and following initiation of treatment for cutaneous disorders.

In conclusion, we have demonstrated that significant age-related differences in hydrophilic mediators exist on the skin of healthy human subjects, corresponding with significant age-based differences in barrier function and skin redness. We also utilized a noninvasive sample collection process. This method is an exciting platform from which more in-depth studies can be executed, providing a clinically relevant means for studying the mechanisms of immunosenescence. All of the detected parameters (TEWL, a(*), and CCBAs) likely relate or contribute to skin cellular senescence. This suggests that there is low level chronic inflammation in aging skin. It also suggests that the presence of inflammatory cytokines may regulate dermal homeostatic events. It is premature but tempting to speculate that there may be also differences in CCBAs, TEWL, and a(*) in local skin lesions of both young and elderly subjects with a variety of dermatologic diseases (rashes, pruritic regions, etc.) correlating with clinical symptoms and pathologies. By including additional aging markers, immunohistochemical analysis, and characterization of senescence-associated secretory phenotypes, we should be able to identify specific phenotypes of aging skin based on barrier function, skin color, hydration, tissue markers of aging, and CCBA profiles.

## Materials and Methods

### Clinical study procedures

This study was approved by the University of Iowa Institutional Review Board and carried out in accordance with the principles set forth by the Declaration of Helsinki. Studies took place in the Clinical Research Unit at the University of Iowa Hospitals and Clinics. Healthy human volunteers between 18–95 years of age with no history of dermatologic disease were recruited. Subjects with clinical conditions that could affect the skin’s healing properties or surface analytes were excluded, and subjects who were taking medications that could affect the skin’s general properties were also excluded. Exclusion criteria included the following: severe general allergies; allergy or adverse reaction to medical tape or adhesive; allergy to latex or rubber; recent sunburn; use of exfoliating dermatologic products; diabetes mellitus; HIV/AIDS; pregnant/nursing; immunologic diseases. Subjects using medications in the following therapeutic classes were also excluded: HMGCoA reductase inhibitors (“statins”); oral or topical steroids; and oral or topical antibiotics (as seen in Supplementary Table S1).

After providing informed consent, baseline demographic information was collected from each subject, including age, sex, height, weight, and information regarding current medical conditions and medications. Three sites were identified on the upper arm and marked with a pen. Three measurements of transepidermal water loss and tristimulus colorimetry were made at each site (methods described below). Following baseline measurements, skin wash fluid was collected from each site for analysis of analytes (methods described below).

There were a total of three sample collection sites in each subject, which allows for a larger number of samples to be assessed within a small sample.

### Transepidermal water loss (TEWL)

Barrier function of the skin was measured in triplicate at each site using a single probe open-chamber evaporimeter (CyberDERM Inc., Broomall, PA). The probe was placed on the skin surface for approximately one to two minutes until the reading stabilized; measurements were recorded in units of g∙m^-2^∙h^-1^ (calculated by the software).

### Tristimulus colorimetry

Skin erythema was quantified via tristimulus colorimetry according to previously published guidelines^7^. The a(*) value represents the red/green axis, and increasing redness results in higher a(*) values. A(*) was measured at each site using a ChromaMeter CR-400 (Konica Minolta, Ramsey, NJ), which was calibrated daily using a white plate provided by the company. To make measurements, the head of the instrument was placed gently on the skin to record the color reflectance; measurements were made in triplicate at each site.

### Collection and analysis of chemokines, cytokines, and biologic analytes from the skin

Skin analytes were collected from skin wash fluid with a simple and noninvasive collection method adapted from previous reports^20^. Small rubber chambers were secured with medical tape (Tegaderm™, 3M) at each of the three sites on the upper arm. The chambers were filled with 500 μl of USP grade sterile phosphate-buffered saline (PBS), pH 6.6–7.2 (Amresco LLC, Solon, OH). After a 30 minute incubation period the PBS was collected, treated with protease and metalloproteinase inhibitors (Fisher Scientific, Waltham, MA) per manufacturer instructions, filtered through a 0.22 μm filter and stored at −80 °C until analysis.

The presence and concentrations of CCBAs were analyzed using multiplexed fluorescent bead-based immunoassays (EMD Millipore, Billerica, MA). The SKINMAG-50K Immune Response Multiplex Assay kit was used to detect and quantify cortisol, fibronectin, involucrin, keratin-6, keratin-1_10, HSA, and LPS. The HCYTOMAG-60K Immunology Multiplex Assay was used to detect and quantify VEGF, sCD40L, EGF, eotaxin, FGF-2, Flt-3 ligand, fractalkine, G-CSF, GM-CSF, GRO, IFN-α2, IFN-γ, IL-1α, IL-1β, IL-1ra, IL-2, IL-3, IL-4, IL-5, IL-6, IL-7, IL-8, IL-9, IL-10, IL-12 (p40), IL-12 (p70), IL-13, IL-15, IL-17A, IP-10, MCP-1, MCP-3, MDC(CCL22), MIP-1α, MIP-1β, PDGF-AA, PDGF-AB/BB, RANTES, TGF-α, TNF-α, and TNF-β.

### Statistical analysis

All data were log-transformed to make a normality assumption more defensible. An analogous two-way fixed effects ANOVA was fit to mediator concentration, TEWL, or a(*) (fixed factors were group and time). Pairwise group comparisons were conducted using the method of Tukey’s Honest Significant Differences. p < 0.05 was considered significant.

## Additional Information

**How to cite this article**: Kinn, P. M. *et al*. Age-dependent variation in cytokines, chemokines, and biologic analytes rinsed from the surface of healthy human skin. *Sci. Rep.* **5**, 10472; doi: 10.1038/srep10472 (2015).

## Supplementary Material

Supplementary Information

## Figures and Tables

**Table 1:** All cytokines, chemokines, and analytes (CCBAs) detected in skin wash fluid of healthy human subjects ranging from 21–77 years of age.

**Response in pg/ml (range)**	**Number of mediators detected**	**Specific analytes**	**Class of analytes**
Negligible (<1.0)	24	GM-CSF, TGFα, PDGF-AA	Growth and stimulating factors
		IL-6, IL-9, TNFα	Pro-inflammatory cytokines
		IL-10, IL-13	Anti-inflammatory cytokines
		IL-8, MCP-1 (CCL2), MIP-1α (CCL3), MIP-1β (CCL4), RANTES (CCL5)	Chemokines
		IL-2, IL-3, IL-4, IL-5, IL-7, IL-12p40, IL-12p70, IL-15, IL-17a, LPS, IFNγ	Misc. analytes
Low (1.0–10.0)	8	EGF*, G-CSF, PDGF-BB	Growth and stimulating factors
		TNFβ	Pro-inflammatory cytokines
		IL-1β, Flt-3L, IFNα2*, GRO	Misc. analytes
Moderate (10.0–100.0)	9	FGF-2*, VEGF	Growth and stimulating factors
		sCD40L	Pro-inflammatory cytokines
		MCP-3 (CCL7), eotaxin (CCL11), MDC (CCL22), Fractalkine (CX3CL1), IP-10 (CXCL10)	Chemokines
		Fibronectin	Misc. analytes
Robust (>100.0)	7	IL-1α	Pro-inflammatory cytokines
		Keratin-1_10, keratin-6*	Keratins
		IL-1RA*, cortisol*, HSA*, involucrin*	Misc. analytes

These classifications are based on mean raw values (not log-transformed data); concentration category was determined by the highest mean value across the three groups. *Denotes the eight analytes that were significantly different between groups, based on log-transformed data.

**Table 2:** Log-transformed concentrations (pg/ml) of the eight CCBAs with significant differences between groups.

**Mediator**	**Group 1**	**Group 2**	**Group 3**
EGF	0.60 ± 0.11	0.10 ± 0.07	0.09 ± 0.05
	(0.0 – 1.36)	(0.0 – 1.2)	(0.0 – 1.04)
	A	B	B
FGF-2	0.48 ± 0.16	0.23 ± 0.11	0.09 ± 0.05
	(0.0 – 1.36)	(0.0 – 1.46)	(0.0 – 0.9)
	A	A,B	B
IFNα2	0.63 ± 0.04	0.46 ± 0.05	0.46 ± 0.04
	(0.34 – 1.04)	(0.0 – 0.89)	(0.00 – 0.86)
	A	B	B
IL-1RA	2.13 ± 0.06	1.44 ± 0.12	1.09 ± 0.10
	(1.84 – 2.64)	(0.06 – 2.5)	(0.00 – 1.98)
	A	B	C
Cortisol	2.53 ± 0.06	2.85 ± 0.10	2.94 ± 0.07
	(2.08 –3.13)	(2.11 – 3.77)	(2.11 – 3.74)
	A	B	B
HSA	4.35 ± 0.06	3.73 ± 0.22	4.02 ± 0.10
	(3.97– 4.98)	(0.00 – 5.00)	(2.53 – 5.02)
	A	B	A,B
Keratin 6	3.44 ± 0.25	1.57 ± 0.41	1.26 ± 0.31
	(0.00 – 4.22)	(0.00 - 4.12)	(0.00 – 3.99)
	A	B	B
Involucrin	1.95 ± 0.10	1.20 ± 0.19	1.18 ± 0.15
	(1.30 – 2.56)	(0.00 – 2.63)	(0.00 – 2.36)
	A	A,B	B
MDC(CCL22)	0.98 ± 0.08	0.85 ± 0.06	0.74 ± 0.07
	(0.00 – 1.40)	(0.34 – 1.29)	(0.00 – 1.23)
	Trending towards significance		
VEGF	0.58 ± 0.13	0.32 ± 0.12	0.24 ± 0.08
	(0.00 – 1.98)	(0.00 – 1.65)	(0.00 – 1.57)
	Trending towards significance		

All data are displayed as mean ± standard error, with minimum and maximum values in parentheses. Levels for Groups 1, 2, and 3 not connected by the same letter within each row are significantly different.
