# Supplementary material for: Age-dependent variation in cytokines, chemokines, and biologic analytes rinsed from the surface of healthy human skin
Source: Sci Rep. 2015 Jun 2;5:10472. doi: 10.1038/srep10472 (PMC4451800; doi:10.1038/srep10472)
Supplement: Supplementary Information [file srep10472-s1.pdf]

Supplementary information

Age-dependent variation in cytokines, chemokines, and biologic analytes rinsed from the surface of healthy human skin

Patrick M. Kinn, Grant O. Holdren, Brittney A. Westermeyer, Mousa Abuissa, Carol L. Fischer,

Janet A. Fairley, Kim A. Brogden, Nicole K. Brogden*

*Corresponding author.

Group	N (M/F)	Age, yrs (range)	BMI (range)	Medical conditions (# of subjects)	Medication classes (# of subjects)
1	6<br>(2/4)	24.3 ± 2.8<br>(21 – 27)	25.3 ± 6.5<br>(20.3 – 37.1)	ADD (2)<br>Asthma (2)<br>Migraines (1)	MVI or supplements (2)<br>Stimulants for ADD (2)<br>Contraceptives (3)<br>Antihistamines (2)<br>Albuterol inhaler (2)<br>Antiemetics (1)<br>Nasal steroid (1)
2	8<br>(1/7)	56.6 ± 4.6<br>(52 – 64)	29.2 ± 7.4<br>(21.2 – 41.9)	Mental illness (3)<br>Bipolar disorder (1)<br>Depression/anxiety (2)<br>Asthma (1)<br>Metabolic syndrome (1)<br>GERD (3)<br>OA of the knee (1)<br>Hypertension (1)	MVI or supplements (4)<br>Antidepressants (3)<br>Antipsychotics (1)<br>Anti-diabetics (1)<br>Sleep aids (1)<br>Nasal steroids (1)<br>PPIs (3)<br>NSAIDs (1)<br>Contraceptives (1)<br>Urinary anti-spasmodic (1)<br>Cardiovascular agents (6)<br>Daily aspirin (3)<br>Anti-hypertensives (3)
3	9<br>(5/4)	72.9 ± 3.0<br>(70 – 77)	27.8 ± 4.14<br>(20.8 – 35.6)	Parkinson's Disease (1)<br>Gout (1)<br>Arthritis (4)<br>Hypertension (3)<br>Hypothyroid (1)	MVI or supplements (6)<br>Antidepressants (1)<br>Anti-Parkinsons (1)<br>Anti-gout (1)<br>Non-NSAID analgesics

				Depression (1)<br><br>Atrial fibrillation (2)<br><br>BPH (2)<br><br>Diverticulitis (1)	(2)<br><br>Cardiovascular agents (12)<br><br>Aspirin (5)<br><br>Anti-hypertensives (4)<br><br>Digoxin (1)<br><br>Anti-coagulants (2)<br><br>Anti-BPH agent (2)<br><br>Thyroid replacement (1)

Supplementary Table S1: General demographics of the study groups, including current medical conditions and daily medications. Abbreviations used in the table: ADD = attention deficit disorder; BPH= benign prostatic hyperplasia; GERD = gastroesophageal reflux disease; MVI = multivitamin; NSAID = nonsteroidal anti-inflammatory drug; OA = osteoarthritis; PPI = proton pump inhibitor.

CCBA	Group 1		Group 2		Group 3	
	Mean (pg/ml)	Std error	Mean (pg/ml)	Std error	Mean (pg/ml)	Std error
IL-1α	1079.6	153.2	731.4	101.2	1011.2	115.9
IL-1RA*	160.0	24.9	42.8	6.6	23.8	5.1
IL-1β	2.5	0.7	2.5	0.7	5.4	2.2
IL-2	0.1	0.08	0.03	0.03	0.06	0.03
IL-3	0.09	0.08	0.0	0.0	0.0	0.0
IL-4	1.0	0.7	0.3	0.1	0.1	0.05
IL-5	0.08	0.03	0.05	0.02	0.05	0.01
IL-6	0.3	0.2	0.03	0.03	0.05	0.03
IL-7	0.5	0.2	0.4	0.2	0.2	0.07
IL-8	0.1	0.08	0.0	0.0	0.0	0.0
IL-9	0.1	0.1	0.1	0.1	0.0	0.0
IL-10	0.4	0.3	0.5	0.1	0.3	0.08
IL-13	0.2	0.1	0.2	0.1	0.1	0.05
IL-15	0.2	0.2	0.0	0.0	0.0	0.0
IL-17a	0.2	0.2	0.0	0.0	0.0	0.0
TNFα	0.05	0.05	0.0	0.0	0.0	0.0
TNFβ	2.7	0.3	2.5	0.2	2.2	0.2
TGFα	0.05	0.05	0.0	0.0	0.0	0.0
MCP-1 (CCL2)	0.9	0.4	0.6	0.2	0.5	0.1
MIP-1α (CCL3)	0.1	0.1	0.1	0.1	0.0	0.0
MIP-1β (CCL4)	0.4	0.4	0.0	0.0	0.0	0.0
RANTES (CCL5)	0.7	0.3	0.6	0.2	0.3	0.07

MCP-3 (CCL7)	2.3	0.9	0.9	0.4	1.6	0.7
Eotaxin (CCL11)	9.7	1.1	12.3	3.8	7.4	0.6
MDC (CCL22)	10.5	1.4	7.7	1.2	6.2	0.8
IL-12p40	0.9	0.4	0.6	0.2	0.3	0.1
IL-12p70	0.3	0.2	0.2	0.1	0.04	0.0
Fractalkine (CX3CL1)	34.7	5.5	30.5	5.8	32.5	4.9
IP-10 (CXCL10)	21.0	2.8	13.9	1.3	16.3	1.6
G-CSF	2.1	0.8	0.9	0.3	1.4	0.3
EGF*	5.9	1.6	0.9	0.7	0.7	0.4
VEGF	9.9	5.2	5.2	2.5	2.7	1.3
FGF-2*	12.2	6.0	3.2	1.7	0.7	0.4
GM-CSF	0.2	0.1	0.0	0.0	0.07	0.03
PDGF-AA	0.1	0.1	0.3	0.3	0.0	0.0
PDGF-BB	1.6	1.3	1.3	0.5	0.5	0.3
IFNα2*	3.7	0.5	2.2	0.4	2.2	0.3
IFNg	0.5	0.2	0.3	0.09	0.3	0.07
sCD40L	15.4	7.0	7.0	3.8	9.4	2.6
Flt-3L	4.1	1.0	5.2	0.8	3.2	0.5
GRO	8.6	1.0	7.5	0.6	6.6	0.4
Fibronectin	83.9	8.7	72.4	8.5	70.7	6.8
Involucrin*	128.9	25.2	68.6	26.5	47.7	11.2
LPS	0.0	0.0	0.0	0.0	0.0	0.0
HSA*	27497.8	4951.1	15516.7	5037.3	19880.7	4541.8
Cortisol*	407.8	67.8	1198.6	294.1	1226.7	203.1
Keratin-	955.0	699.9	1455.7	1455.7	1583.7	1583.7

1_10						
Keratin-6*	6676.7	1349.3	2622.4	888.9	1598.0	514.1

Supplementary Table S2. Mean and standard error of the non log-transformed concentrations of CCBAs detected in skin wash fluid across all three subject groups.
*Denotes CCBAs with significant differences between groups.
